# Rich Magnetic Quantization Phenomena in AA Bilayer Silicene

**DOI:** 10.1038/s41598-019-50704-0

**Published:** 2019-10-15

**Authors:** Po-Hsin Shih, Thi-Nga Do, Godfrey Gumbs, Danhong Huang, Hai Duong Pham, Ming-Fa Lin

**Affiliations:** 1 Department of Physics, National Cheng Kung University, Tainan City, Taiwan; 2 Laboratory of Magnetism and Magnetic Materials, Advanced Institute of Materials Science, Ton Duc Thang University, Ho Chi Minh City, Vietnam; 3 Faculty of Applied Sciences, Ton Duc Thang University, Ho Chi Minh City, Vietnam; 4 Department of Physics and Astronomy, Hunter College of the City University of New York, 695 Park Avenue, New York, New York, 10065 USA; 5 Donostia International Physics Center (DIPC), P de Manuel Lardizabal, 4, 20018 San Sebastian, Basque Country Spain; 6 US Air Force Research Laboratory, Space Vehicles Directorate, Kirtland Air Force Base, New Mexico, 87117 USA; 7 Quantum Topology Center, National Cheng Kung University, Tainan City, Taiwan; 8 Hierarchical Green-Energy Materials Research Center, National Cheng Kung University, Tainan City, Taiwan

**Keywords:** Materials science, Physics

## Abstract

The rich magneto-electronic properties of AA-bottom-top (bt) bilayer silicene are investigated using a generalized tight-binding model. The electronic structure exhibits two pairs of oscillatory energy bands for which the lowest conduction and highest valence states of the low-lying pair are shifted away from the K point. The quantized Landau levels (LLs) are classified into various separated groups by the localization behaviors of their spatial distributions. The LLs in the vicinity of the Fermi energy do not present simple wave function modes. This behavior is quite different from other two-dimensional systems. The geometry symmetry, intralayer and interlayer atomic interactions, and the effect of a perpendicular magnetic field are responsible for the peculiar LL energy spectra in AA-bt bilayer silicene. This work provides a better understanding of the diverse magnetic quantization phenomena in 2D condensed-matter materials.

## Introduction

Silicene, an isostructure to graphene, is purely made of silicon atoms through both the *sp*^2^ and *sp*^3^ bondings. So far, silicene systems have been successfully synthesized by the epitaxial growth on various substrate surfaces. Monolayer silicene with different sizes of unit cells has been produced on several substrates, such as Si(111) $\sqrt{3}\times \sqrt{3}$-Ag template^1,2^, Ag(111) (4 × 4)^3,4^, Ir(111) ($\sqrt{3}\times \,\sqrt{3}$)^5^ and ZrB_2_(0001) (2 × 2)^6^. Such a buckled single-layer honeycomb lattice was clearly identified by high-resolution measurements of STM and low-energy electron diffraction. It should be noted that the examined system might present an enlarged unit cell because of the significant effect due to the substrates, e.g., the significant orbital hybridization between silicon atoms and those from the substrate. The energy band shows a Dirac-cone structure with a graphene-like Fermi velocity but in the presence of a small spin-orbital coupling of ~2 meV, as directly confirmed by angle resolved photoemission spectroscopy (ARPES) experiments^3,4^. Bilayer silicene has predicted of being capable of exhibiting four distinct types of stacking configurations, namely AB-bt, AB-bb (bottom-bottom), AA-bt, and AA-bb^7–14^. Experimental fabrication has been reported for the two AB stable bucklings^7^. However, there is still a lack of similar experimental study for AA-stacked bilayer silicene.

Theoretical investigations have been focused on the fundamental properties of monolayer and bilayer silicene with or without adatom chemisorptions or guest atom substitutions based on various approaches, covering the first-principles calculations^8–16^, the generalized tight-binding model^17,18^, and the effective-mass approximation^19^. Whereas the first method is suitable in studying the optimal geometries, the later two are powerful tools for the exploration of magnetic quantization. Nevertheless, the effective-mass model reveals certain limitations in dealing with complicated systems such as ABC trilayer graphene^15^, AB-bt bilayer silicene^17^ and others.

Monolayer silicene has been predicted to possess a relatively narrow gap of ~5 meV^6^, as a consequence of weak spin-orbital coupling. The interplay between intrinsic interactions and electric or magnetic fields may cause the destruction of (*z* = 0)-plane mirror symmetry (electric field) and the appearance of periodic Peierls phases (magnetic field), respectively. The former gives rise to the spin-dominated split energy band and the significant change in band gap^19^ the latter yields highly degenerate Landau levels^17^. Recently, silicene with adatom (X) systems have been investigated for chemical modifications by the remarkable multi-orbital hybridization in Si-Si, Si-X and X-X bonds by VASP calculations^15,17^. Such phenomena might induce metallic or semiconducting behavior in materials. However for bilayer silicene, phenomenological models might not be suitable for solving the magnetic-field-dominated fundamental properties due to significant buckling, the largely enhanced spin-orbital interactions and the complex interlayer hopping integrals. Specifically, optimization of reliable tight-binding parameters which are necessary for reproducing the low-lying two pairs of valence and conduction bands are likely impossible because of the complex features in the first-principle results for the energy dispersion relations^8–14^. Bilayer silicene with AA and AB stackings are predicted to present the non-monotonic energy dispersion and irregular valleys at non-high-symmetry points. Additionally, the free carrier densities are expected to be quite sensitive to buckling and stacking configurations.

In this paper, we explore the diverse quantization phenomena in AA-bt silicene by employing a generalized tight-binding model. Calculations and analysis will target the band properties both below and above the Fermi level, energy dispersion relations, critical points in energy-wave-vector space, distinct valleys, special structures of van Hove singularities in the density-of-states (DOS), significant magnetic-field dependencies, classification of valence and conduction LL groups and their main features. Specifically, the valley-enriched magnetic quantization will be investigated by detailed examination of the spatial oscillation modes of the magnetic wave functions. The interesting combined effects of distinct LL groups are clarified from different stable or metastable valleys. Furthermore, we also discuss the important differences in the essential physical properties between AA-bt bilayer silicene, monolayer silicene and graphene from the electronic valley structure point of view.

## Method

**Figure 1:**
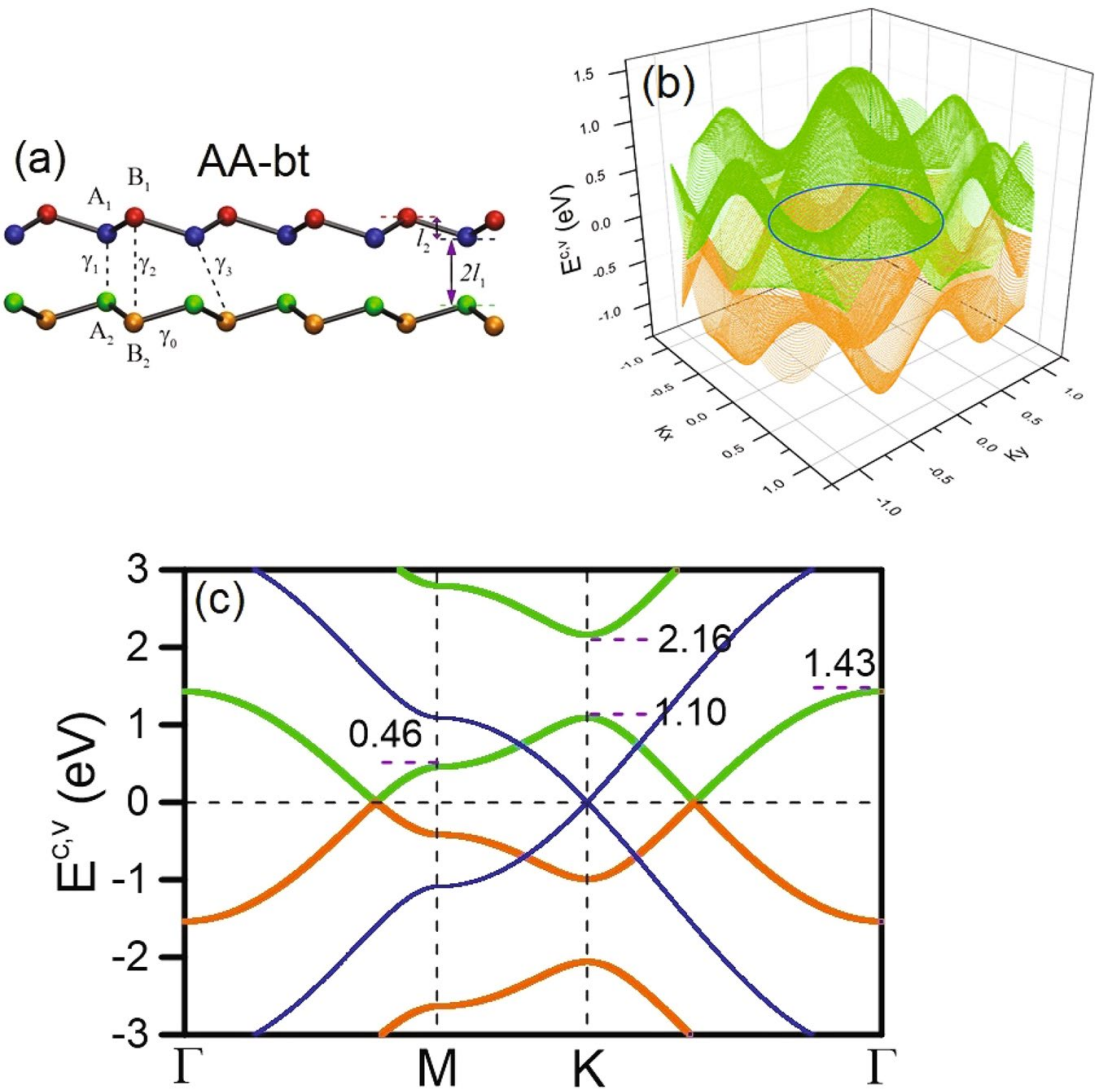
(**a**) Side view of the geometric structure including the significant hopping integrals for AA-bt bilayer silicene. The low-energy band structure is shown for (**b**) 3D and (**c**) 2D along the high symmetry directions. For comparison, the energy band of monolayer silicene is also plotted in (**c**).

We have developed a generalized tight-binding model for AA-bt bilayer silicene in the presence of a uniform perpendicular magnetic field and utilized it to explore the magnetoelectronic properties. The crystal structure of AA-bt bilayer silicene with hopping interaction terms is clearly illustrated in Fig. 1(a). The two layers possess opposite buckling order, in which the [A^1^, A^2^] sublattices lie in the inner planes while the [B^1^, B^2^] sublattices are located at the outer planes. The lattice constant and bond length are *a* = 3.83 *Å* and *b* = 2.21 *Å*, respectively. A primitive unit cell contains four silicon atoms. Accordingly, the critical Hamiltonian is built from the four tight-binding functions of Si-3p_*z*_ orbitals. It can be written as

$$H=\sum _{i,l}\,{U}_{i}^{l}{c}_{i}^{\dagger l}{c}_{i}^{l}+\sum _{\langle i,j\rangle ,l,l\text{'}}\,{\gamma }_{ij}^{ll\text{'}}{c}_{i}^{\dagger l}{c}_{j}^{l\text{'}}.$$

In this notation, ${c}_{i}^{\dagger l}$ (${c}_{i}^{l}$) is the creation (annihilation) operator which could generate (destroy) an electronic state at the *i*-th site of the *l*-th layer. ${U}_{i}^{l}$(*A*^*l*^, *B*^*l*^) is the buckled-sublattice-height-dependent Coulomb potential energy due to the applied gate voltage. ${\gamma }_{ij}^{ll\text{'}}$ represents the intra- and inter-layer atomic interactions, in which the former comes from the nearest-neighbor interactions in the [*A*^*l*^, *B*^*l*^] sublattices (*γ*_0_ = 1.004 eV) and the latter stand for interactions between sublattices from different layers (*γ*_1_ = −2.110 eV, *γ*_2_ = −1.041 eV, and *γ*_3_ = 0.035 eV), as shown in Fig. 1(a). These parameters are optimized using the Slater–Koster tight-binding method in order to reproduced the energy bands from the first-principle results^9,11^. Interestingly, our optimization shows that *γ*_1_ is much larger than when *γ*_2_ is comparable to *γ*_0_. In addition to the high symmetry of stacking configuration, this result might be responsible for the negligible spin-orbital couplings. It seems that in the absence of spin-orbital couplings, the above Hamiltonian is sufficient for the investigation of certain essential physical properties.

The application of a uniform perpendicular magnetic field evidently changes the main characteristics of the lattice. The original unit cell is considerably enlarged to become a long rectangular due to the field-induced extra Peierls phases^17^. The extended unit cell includes 16*ϕ*_0_/*ϕ* Si atoms, where *ϕ*_0_ = *hc*/*e* is the magnetic flux quantum and $\varphi ={B}_{z}\sqrt{3}{a}^{2}/2$ is the magnetic flux through a unit cell. Consequently, the magnetic Hamiltonian is a huge Hermitian matrix, e.g., the size is ~13000 × 13000 for a (*k*_*x*_ = 0, *k*_*y*_ = 0) state at *B*_*z*_ = 20 T. For AA-bt bilayer silicene, the model calculation takes into account the buckled honeycomb structure and complicated intra- and inter-layer atomic interactions. The combined effect of those ingredients and an external magnetic field is expected to generate the diverse physical phenomena, especially the magneto-electronic properties.

## Results and Discussion

### Zero-field electronic properties

**Figure 2:**
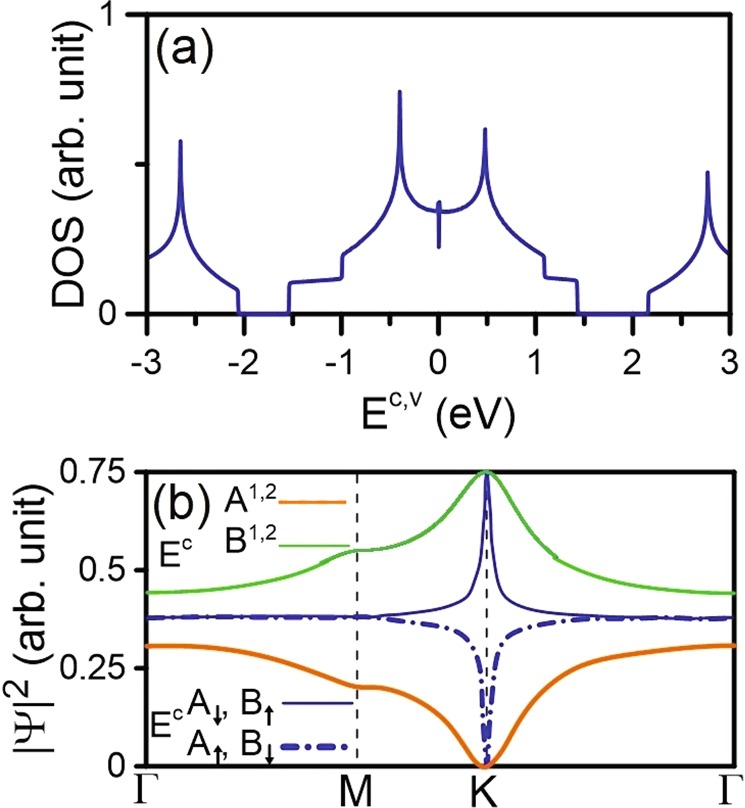
The (**a**) van Hove singularities in density of states. The wave-vector-dependent wave functions are shown in (**b**). The solid and dashed blue curves of the A^1^ and B^1^ sublattices, respectively.

AA-bt bilayer silicene presents the unique and feature-rich energy dispersion, as demonstrated in Fig. 1(b,c) for 3D and 2D views, respectively. There are two pairs of valence and conduction bands due to the Si-3*p*_*z*_-orbital *π* bondings and they are slighly asymmetric about the Fermi level of *E*_*F*_ = 0. It shows the semimetallic behavior with zero gap and a finite density of states (DOS) at *E*_*F*_, as clearly illustrated in Fig. 2(a). The outer pair of conduction and valence energy bands is originated at higher and deeper energy ranges (|*E*^*c*^, *v*| ≥ 2 eV). On the other hand, the pair of energy bands near *E*_*F*_ is expected to dominate the low-energy essential properties of the system. Interestingly, the stable and non-stable electronic valleys are formed from the electronic states near the high-symmetry points of the hexagonal first Brillouin zone. In particular, there exist the M, K and Γ valleys with different conduction and valence band-edge state energies of about (0.5 eV, −0.51 eV), (1.19 eV, −1.21 eV), and (1.43 eV, −1.50 eV), respectively. These special valleys are expected to be closely related to the magnetic quantizations of the initial Landau levels, as discussed later.

The M valley belongs to the saddle point while the K and Γ valleys correspond to the local extreme points, therefore they lead to the different van Hove singularities (Fig. 2(a)) and the diverse LL energy spectra. Furthermore, the Dirac-cone energy dispersion is absent because the low-lying electronic states closest to the Fermi level are not formed at K/K′ valleys. Instead of upward conduction and downward valence Dirac cone initiated from the K/K′ points as in monolayer graphene^20^, there exist the concave-downward conduction and concave-upward valence valley in AA-bt bilayer silicene. Such a significant difference between the two system might come from the more complicated and stronger atomic interactions of the latter. It should be noticed that, the lowest conduction and highest valence electronic states in AA-bt stacking are located at the midway of M and Γ points, moreover, they are separated by a very small energy spacing of ~8 meV. As a result, the density of states near *E*_*F*_ present the special structure with a finite value. Moreover, the low-lying Landau levels do not correspond to the initial magneto-electronic states. In general, the electronic properties in AA-bt bilayer silicene are in great contrast with those of monolayer silicene^4^ and a AA-stacked bilayer graphene^21^. The van Hove singularities in the DOS are closely associated with three types of band-edge states, as illustrated in Fig. 2(a). The DOS spectrum exhibits feature-rich structures, including asymmetric peaks in the square-root divergent form, shoulder structures, and logarithmically divergent peaks. There exists a pair of temple-like cusp structures crossing the Fermi level and they are separated by quite a small energy spacing of ~8 meV. These structures come from the extraordinary conduction and valence band structures near *E*_*F*_ which could be considered as the one-dimensional parabolic dispersion relations. Away from the Fermi level, the two pairs of prominent symmetric peaks arising from the 2D saddle point (M point) are located at (0.50 eV & −0.52 eV) and (2.60 eV & −2.65 eV), respectively. Regarding the extreme points, there appear special shoulder structures at (1.10 eV, −1.15 eV) and (2.16 eV, −2.20 eV) for the K valley and (1.43 eV, −1.5 eV) for the Γ one. The special DOS spectrum in AA-bt bilayer silicene reflects the unique energy dispersion, it could be examined from high-resolution STS experiments^22–25^. This method of measurement is useful for investigating the interplay between the buckled structure and atomic interactions in bilayer silicene. For AA-bt stacking, the DOS is relatively high near the M point compared with other high-symmetry points, leading to the very complex LL energy spectrum, dissimilar to those of monolayer graphene and silicene^19,26^. Roughly speaking, the M valley is regarded as the unstable one for magnetic quantization from where LLs could not be initiated.

The Bloch wave functions consisting of the Si-3*p*_*z*_-orbital tight-binding functions on the four sublattices strongly depend on the wave vectors, as clearly shown in Fig. 2(b). Those on the A and B sublattices are represented by orange and green curves, respectively. For comparison, the result for monolayer silicene is also shown as solid and dashed blue curves. The low-lying conduction band (and valence band; not shown) exhibit equal distribution probability on the B^1^ & B^2^ [A^1^ & A^2^] sublattices due to the same (*x*, *y*)-plane projections with similar chemical environment. Specifically, electronic states near the K point exhibit the vanishing A^1^ and A^2^ components and the identical B^1^ and B^2^ ones. Along the K → M → Γ directions, the B^*l*^- and A^*l*^-sublattice probabilities vary as 0.5 → 0.375 → 0.265 and 0 → 0.125 → 0.2355, respectively. Such behavior is quite different from that in monolayer silicene where the distribution of all sublatices become identical along M → Γ. Generally, the distribution probability of B^*l*^ sublattices is evidently dominated by the low-lying energy spectrum. The opposite is true for the higher conduction and deeper valence bands. That is, the equivalence of the intralayer [*A*^*l*^, *B*^*l*^] sublattices is completely broken by the strong interlayer hopping integrals (*γ*_1_ and *γ*_2_). On the other hand, monolayer silicene with significant spin-orbital coupling present similar distribution probabilities of the sublattices of the same spin state during variation of the wave vector due to the honeycomb lattice symmetry.

### Diverse magnetic quantization phenomena

AA-bt stacking possesses distinctive and diverse magnetic quantization phenomena, mainly owing to the feature-rich valley structures of the low-lying pair of conduction and valence bands. The LL energy spectra exhibit a number of interesting characteristics, These include LL state degeneracy, localization behavior of wave functions, sublattice dependence of localized oscillation modes, well-behaved and not well-behaved LLs, complex magnetic field dependence, and LL crossing phenomenon. It is worth mentioning that the localization centers of LLs are continuously changed with the variation of (*k*_*x*_, *k*_*y*_). Similar phenomena are observed for different electronic states. Our numerical calculations in this work mainly focus on the magnetic quantization at (*k*_*x*_ = 0, *k*_*y*_ = 0) state, which is sufficient in understanding the essential magneto-electronic properties. The LLs could be classified into different groups based on the electronic valleys and the main features of spatial distributions.

**Figure 3:**
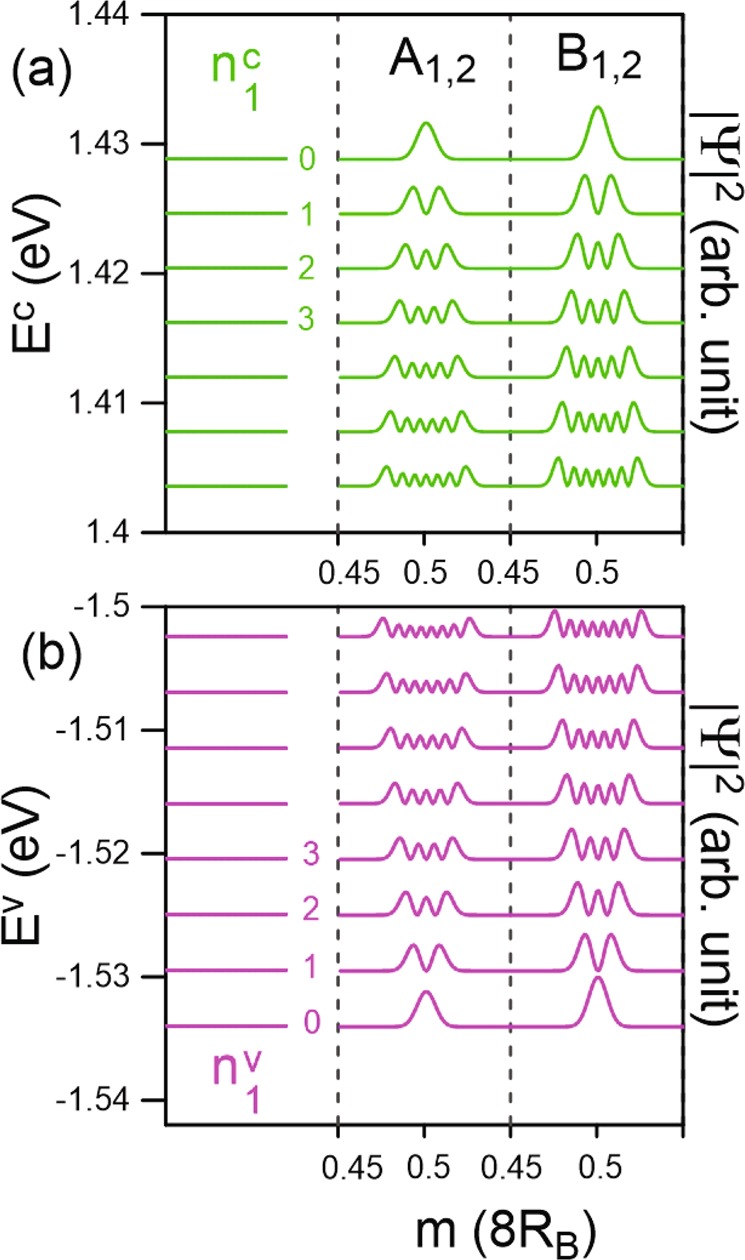
The Landau levels and wave functions when *B*_*z*_ = 40 T for the (**a**) conduction and (**b**) valence bands of the first group.

Regarding the LLs initiated from the Γ-point top and bottom valleys, the conduction and valence energy spectra present asymmetry behavior explicitly. The conduction and valence Landau levels are, respectively, located at 1.43 eV and −1.53 eV for *B*_*z*_ = 40 T, as shown in Fig. 3(a,b). The LLs exhibit a nearly uniform energy spectrum, as a result of the isotropically parabolic energy dispersion near the Γ point, similar to that for a 2D electron gas^27,28^. The well-behaved magnetic subenvelope functions on the four sublattices are localized at 0 and 1/2 of the *B*_*z*_-enlarged unit cell, and they are degenerate. The oscillation modes of the LLs on all four subenvelope functions are equivalent. Additionally, the LL wave functions on the (A^1^ & A^2^) as well as (B^1^ & B^2^) sublattices are observed to be identical. These special characteristics of the LLs may be related to the equivalence of the intralayer [A^*l*^, B^*l*^] sublattices and the same chemical environment for the interlayer [A^1^, A^2^] & [B^1^, B^2^] sublattices. The quantum number of each LL, ${n}_{1}^{c,v}$, is determined by the number of zero modes of its wave function on the dominated B_*i*_ sublattices. The fact that the B^*l*^ sublattices clearly dominate the energy spectra at the Γ valley is consistent with the zero-field wave vector-dependent wave functions as shown in Fig. 2(b). In general, each LL is four-fold degenerate through the spin and localization degrees of freedom. This is dissimilar to the eight-fold degeneracy of the LLs in monolayer graphene and silicene^19,26^.

**Figure 4:**
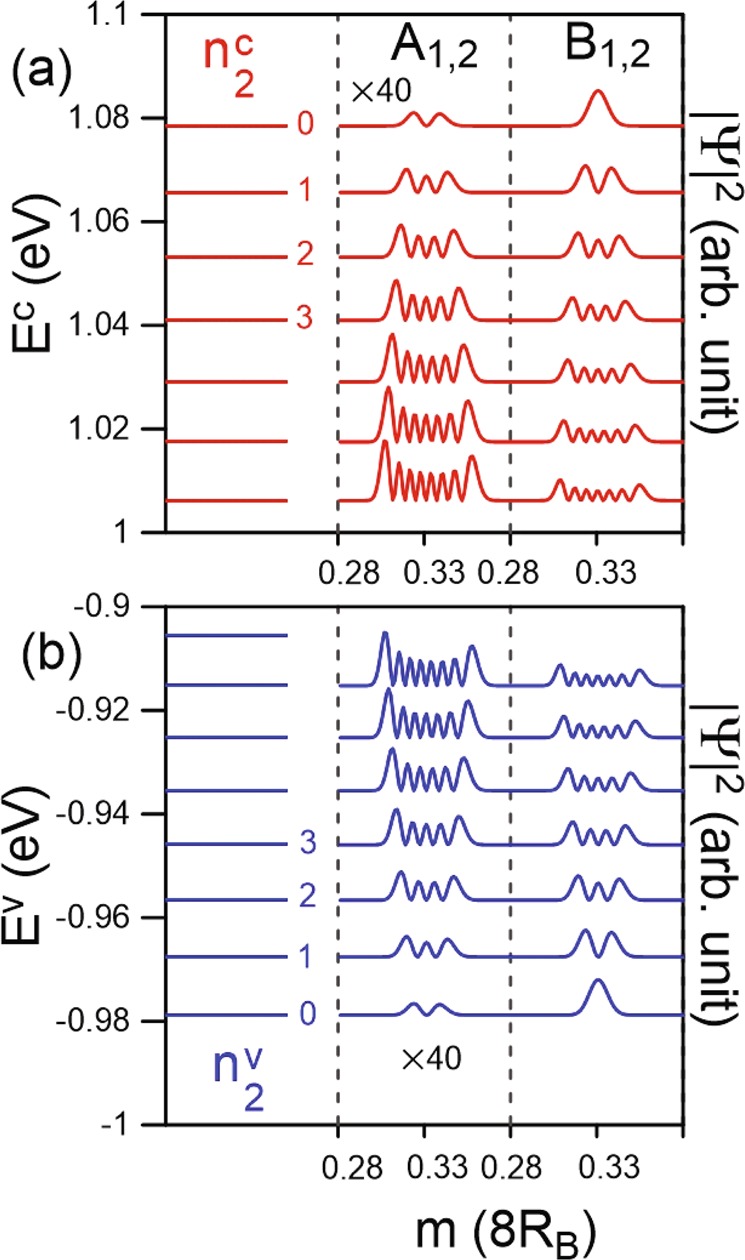
The Landau levels and wave functions at *B*_*z*_ = 40 T for (**a**) the conduction and (**b**) the valence bands of the second group.

The conduction and valence Landau levels magnetically quantized from electronic states near the K/K′ points display diverse characteristics. However, there are LLs corresponding to both the low-lying and outer pairs of energy bands. The former are of central interest, as illustrated in Fig. 4(a,b). Although the LL energy spectrum presents roughly uniform spacing, similar to that near the Γ valley, the LLs show quite different behaviors. The magnetic subenvelope functions are revealed at four distinct localization centers (1/6, 2/6 4/6 and 5/6) of the extended unit cell. Especially, the LL spatial distributions are identical for 1/6 and 4/6 as well as 2/6 and 5/6 areas, leading to eight-fold degeneracy of the LLs, as observed in monolayer graphene^24^. The first few LLs are well-behaved and their quantum numbers (${n}_{2}^{c,v}$) could be determined based on the wave functions of the evidently dominated *B*^*l*^ sublattices. The spatial distributions on the A^*l*^ and B^*l*^ sublattices exhibit one-mode difference, in which those on B^*l*^ is higher (for 1/6 center) or less (for 2/6 center) than the corresponding ones on A^*l*^, depending on the localization centers. Apparently, the initial ${n}_{2}^{c,v}$ = 0 LLs at 2/6 center are exceptionally four-fold degenerate because such magneto-electronic states only originate from the B^*l*^ sublattices.

Away from the initiated level, LLs which are quantized from Γ and K valleys are slightly distorted because of the strong interlayer atomic interactions (*γ*_1_ and *γ*_2_), leading to difficulty in defining their quantum numbers. Regarding the ${n}_{1}^{c,v}$ LLs, although their oscillation distributions become much wider, which are ~35% of a unit cell, they still remain two degenerate localization centers of 0 and 1/2. This further illustrates the monotonic variation of the stable Γ valley along the various directions. For the ${n}_{2}^{c,v}$ LLs, the splitting of LL states at different localization centers of (1/6, 2/6, 4/6, and 5/6) is no longer observable. Their quantum numbers are expected to be much smaller than those of the ${n}_{1}^{c,v}$ group due to the higher DOS in the K-related valleys (Fig. 2(a)). Additionally, the magnetic quantization demonstrates the fact that the M-point saddle structure belongs to the K/K′ valley.

**Figure 5:**
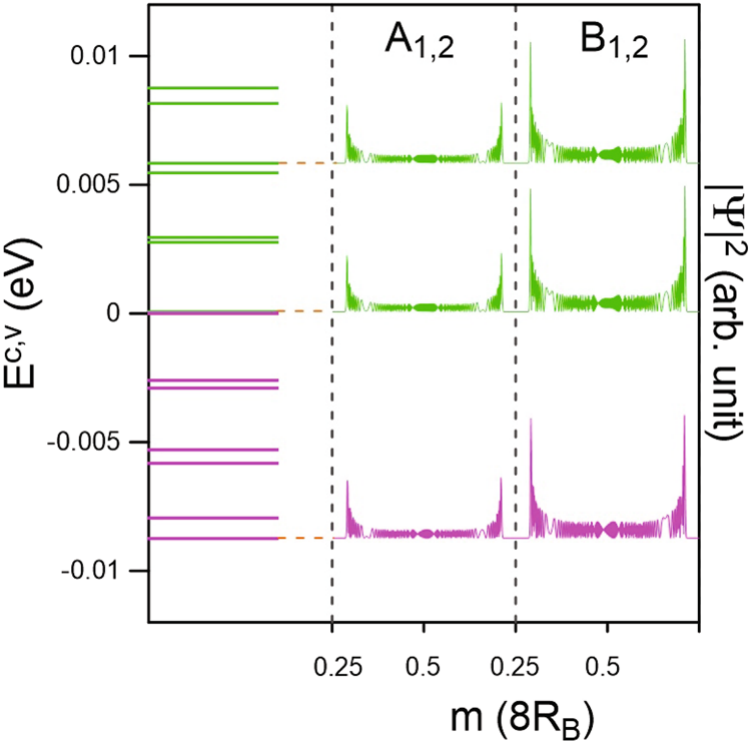
The Landau levels and wave functions at *B*_*z*_ = 40 T of the first group near the Fermi energy. The conduction and valence LLs are presented in green and pink, respectively.

In the vicinity of the Fermi level, there exist only conduction and valence LLs which are quantized from the Γ valley, as clearly shown in Fig. 5. Here, the magneto-electronic states are quite dense and complicated. Therefore, it is quite difficult to characterize the LLs. The LL spatial distributions present thousands of oscillation modes which are localized at 0 and 1/2 centers. Such unique magnetic quantization phenomena are never observed in other condensed-matter systems according to prior theoretical and experimental studies^17,19,26^. Apparently, high-resolution STS measurements^23–26^ may not be able to directly identify the low-energy LLs in AA-bt bilayer silicene. Nevertheless, the array of low-energy magneto-electronic states in AA-bt bilayer silicene should be associated with the other essential physical properties, such as, the delta function-like van Hove singularities, magneto-optical absorption spectra with specific selection rules, quantum Hall transport and inter-Landau level damping and magnetoplasmon modes. These should be worthy of a systematic investigation despite possibly formidable challenges to be encountered in the numerical technique and detailed data analysis.

**Figure 6:**
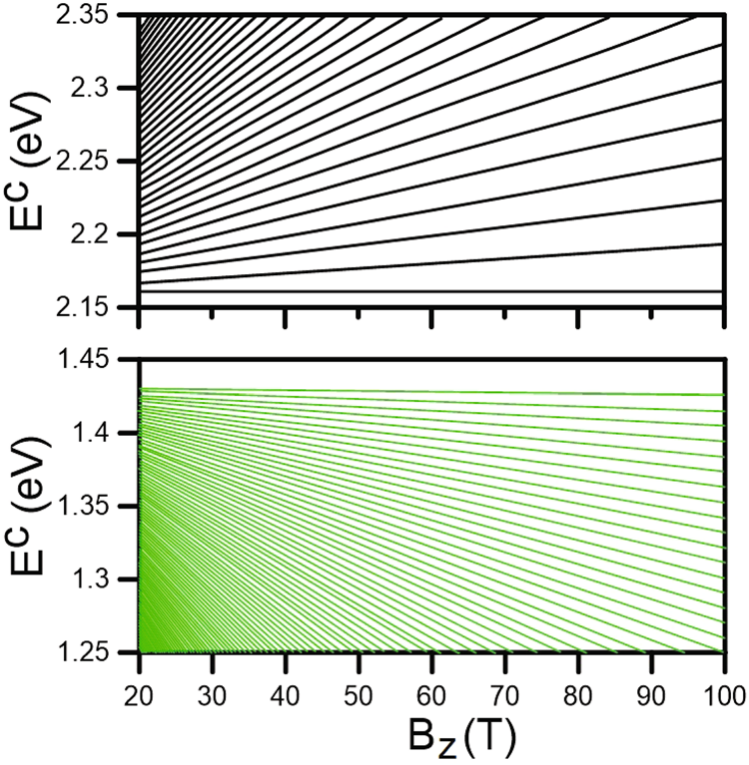
The magnetic field-dependent conduction Landau level energy spectra for (**a**) the first (green curves) and (**b**) third (black curves) groups.

**Figure 7:**
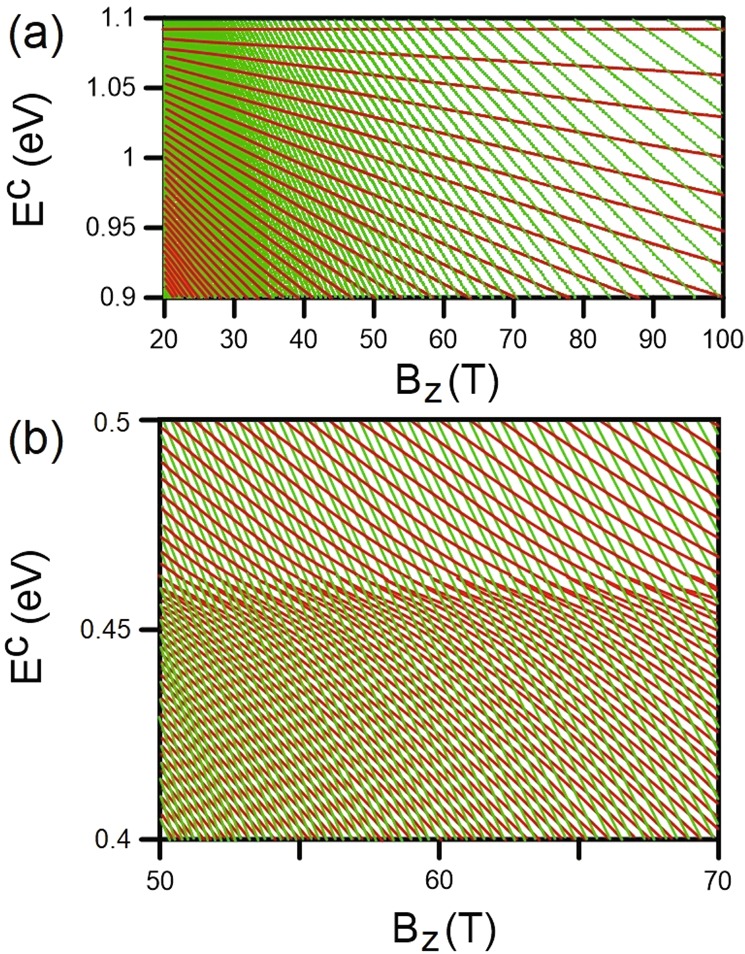
The magnetic field-dependent conduction Landau level energy spectra for the first (green curves) and second (red curves) groups at (**a**) higher and (**b**) lower energy ranges.

The magnetic field-dependent energy spectra are critical for the particularized comprehension of the magnetic quantization phenomena. Although the conduction and valence LLs possess asymmetric spectra, their main features are similar. Therefore, the following discussion will be confined to the conduction bands. The quantized LLs of the outer pair of energy bands show linear *B*_*z*_-dependence, as demonstrated in Fig. 6(a) for the conduction spectrum. The split energy of neighboring LLs gradually increases with the growth of field strength. These quantization characteristics indicate the stable K/K′ valleys for such electronic structure. On the other hand, the dependence of LL groups corresponding to the low-lying energy bands on the variation of magnetic field is much more complicated. The energy spectra are a combination of both ${n}_{1}^{c,v}$ and ${n}_{2}^{c,v}$ LL groups with frequent intragroup and intergroup crossing behaviors, as illustrated in Figs 6(b) and 7(a,b).

We now turn our attention to the ${n}_{1}^{c}$ LLs originating from the Γ point. The initiated LLs are linearly dependent on *B*_*z*_ without any intragroup crossing (Fig. 6(b)), like that for the outer energy bands but with smaller LL energy spacing. With decreasing energy, the *B*_*z*_-dependent spectrum consists of both the ${n}_{1}^{c}$ (green curves) and ${n}_{2}^{c}$ (red curves) LLs, as shown in Fig. 7(a,b). Interestingly, the two groups of LLs present crossing phenomena without any hybridization of magneto-electronic states. This is because the two stable valleys of K and Γ are independent of each other. The evolution of ${n}_{2}^{c}$ LLs with an increase of magnetic field strength has a simple linear behavior for sufficiently high energy, *E*^*c*^ ≥ 0.5 eV, as clearly demonstrated in Fig. 7(a). In contrast, LLs at *E*^*c*^ ~ 0.45 eV are formed with a shoulder-like structure, corresponding to the energy dispersion around the M point. The main reason for this is that the electronic states at very high DOS cannot be quantized into the well-behaved LLs. It is worthy mentioning that, the magnetic field-dependent energy spectra enables the prediction of LL characteristics at different field strengths when those at a specific *B*_*z*_ are known. Since there is no interaction (anti-crossing) between the levels for the variation of *B*_*z*_, the LL wavefunctions remain unchanged.

The magneto-electronic properties of an AA-bt bilayer system are in great contrast with those in monolayer silicene and graphene^19,26^. Whereas the former shows initial LLs at higher and lower energy ranges, those of the latter begin near *E*_*F*_ = 0. Apparently, the main differences lie in the main characteristics of the LLs around the Fermi level which are very complicated for AA-bt stacking. Monolayer systems possess the conduction and valence Dirac cones with the Dirac point or extremely narrow gap at the K/K′ point. The low-lying LLs present well-behaved spatial distributions and therefore their quantum numbers are easily determined based on the number of zero modes. Moreover, the *B*_*z*_ dependence of LL energies exhibit linear form in the absence of crossing phenomenon, unlike the frequent crossing LLs in AA-bt stacking. The above-mentioned diverging points of AA-bt bilayer silicene compared with other monolayer systems mainly come from its unique intrinsic properties, such as the buckling structure with opposite ordering and significant interlayer atomic interactions.

## Conclusion

We have presented a comprehensive investigation of the unusual and diverse magnetic quantization phenomena in AA-bt bilayer silicene using a generalized tight-binding model. This material possesses special electronic structure with two pairs of energy bands, in which the low-lying pair shows an interesting oscillatory shape. Remarkably, the lowest conduction and highest valence states near the Fermi level are away from the K point, dissimilarly to that for graphene and other 2D materials. The intricate energy dispersion is closely related to the feature-rich magnetically quantized LLs. These LLs originating from tje K and Γ valleys are quite different in their main characteristics, covering the LL degeneracy, spatial distributions, the dominant sublattices, and the magnetic field dependence. Specifically, in the vicinity of the Fermi energy, there are many magneto-electronic states which do not present simple wave functions. The geometry symmetry, intralayer and interlayer atomic interactions, and effect of a perpendicular magnetic field are responsible for the peculiar LL energy spectra in AA-bt bilayer silicene.
